# Measurement and visualization of cell membrane surface charge in fixed cultured cells related with cell morphology

**DOI:** 10.1371/journal.pone.0236373

**Published:** 2020-07-23

**Authors:** Masaru Nishino, Ibu Matsuzaki, Fidele Y. Musangile, Yuichi Takahashi, Yoshifumi Iwahashi, Kenji Warigaya, Yuichi Kinoshita, Fumiyoshi Kojima, Shin-ichi Murata

**Affiliations:** Department of Human Pathology, Wakayama Medical University, Wakayama, Japan; CIC bioGUNE, SPAIN

## Abstract

The diagnosis of patients with malignancies relies on the results of a clinical cytological examination. To enhance the diagnostic qualities of cytological examinations, it is important to have a detailed analysis of the cell’s characteristics. There is, therefore, a need for developing a new auxiliary method for cytological diagnosis. In this study, we focused on studying the charge of the cell membrane surface of fixed cells, which is one of important cell’s characteristics. Although fixed cells lose membrane potential which is observed in living cells owing to ion dynamics, we hypothesized that fixed cells still have a cell membrane surface charge due to cell membrane components and structure. We used 5 cell lines in this study (ARO, C32TG, RT4, TK, UM-UC-14). After fixation with CytoRich Red, we measured the cell membrane surface charge of fixed cells in solution using zeta potential measurements and fixed cells on glass slides, visualizing it using antibody-labeled beads and positively-charged beads. Furthermore, we measured the cell membrane surface charge of fixed cells under different conditions, such as different solution of fixative, ion concentration, pH, and pepsin treatments. The zeta potential measurements and visualization using the beads indicated that the cell membrane surface of fixed cells was negatively charged, and also that the charge varied among fixed cells. The charge state was affected by the different treatments. Moreover, the number of cell-bound beads was small in interphase, anaphase, and apoptotic cells. We concluded that the negative cell membrane surface charge was influenced by the three-dimensional structure of proteins as well as the different types of amino acids and lipids on the cell membrane. Thus, cell surface charge visualization can be applied as a new auxiliary method for clinical cytological diagnosis. This is the first systematic report of the cell membrane surface charge of fixed cells.

## Introduction

The diagnosis of patients with malignancies relies on the results of a cytological examination, which constitutes the most important part of the patients’ clinical examination. A cytological examination of cytology specimens distinguishes between benign and malignant cells, based on the presence of cellular atypia such as irregular cell shape, abnormal cytoplasm, nuclear swelling with irregular contour and hyperchromatism [1]. Although these biological cellular characteristics are important, the cell membrane surface, which has been so far overlooked, may also be a part of the abnormal cytological findings in a cytological examination. Generally, abnormal nuclear and cytoplasmic findings are associated with cell proliferation [1] and cell differentiation [1], respectively. However, the cell membrane surface is also important for cell adhesion, function, differentiation, and cell division.

To assess abnormalities of the cell membrane surface, we studied the cell membrane surface charge. The surface charge can have either a negative or positive electrical state, which is determined by the balance between negatively charged and positively charged nanoparticles at the surface. The cell membrane surface of living cells has a different electric potential to the interior of cell, namely membrane potential. The membrane potential of resting cells is usually negative. The membrane potential has been studied well and it has been confirmed that intracytoplasmic materials including ions, sugars, proteins, lipids, etc. affect the membrane potential in living cells [2–6]. For example, Paramecium moves its motile cilia and propels itself through the water by changing its membrane potential [7, 8]. In human cells, it is well known that a membrane potential change affects the information transmission and excitation of living nerve cells and cardiomyocytes [9]. However, a membrane potential is only found in living cells and cannot be detected in fixed cells in cytological specimens.

Although fixed cells do not have a membrane potential, they can have some charge on their cell membrane surface, which is related with cellular membrane composition. This fact has well studied in living bacterial cells, however, few studies for fixed eukaryotic cells were reported [10–12]. Some of the cytology specimen preparation methods for liquid-based cytology (LBC), apply negative charge on the cell membrane surface of fixed cells to attach fixed cells on the positively-charged glass slides [13]. Except for this application for LBC, there are no systematic studies of the membrane surface charge of fixed cells. It is possible that the cell membrane surface charge of fixed cells is related to components of the cell membrane, such as sugars, proteins, and lipids. Therefore, the cell membrane charge may differ among cell types, benign or malignant cells, or differentiation states. In this study, we aimed to study the cell membrane surface charge of fixed cultured cells. To the best of our knowledge, this is the first systematic report of the cell membrane surface charge of fixed cells.

## Materials & methods

### Cell culture

We used 5 different cell lines (ARO, C32TG, RT4, TK and UM-UC-14) in this study. Table 1 shows the details of the cell lines with a brief description of the culture conditions used, according to the instructions from the cell banks. All the cells were fixed using CytoRich Red (Becton Dickinson, Franklin Lakes, NJ) for 24 hours or more, at room temperature. To study the influence of different fixatives to the cell membrane surface charge, TK cells were also fixed with 10% formalin, Carnoy’s fixative composed of methanol and acetic acid (3:1) and PreservCyt Solution (Hologic Japan, Tokyo, Japan). After fixation, the cells were washed 2 times with phosphate buffered saline (PBS) (NaCl 1370 mM, KCl 27 mM, Na2HPO4112H2O 81 mM, KH2PO4 18 mM).

**Table 1:** Characteristics of the cell lines used and culture conditions.

Name	Derived from	Culture conditions	Cell bank
ARO	Undifferentiated carcinoma of thyroid	• DMEM + High Glucose + GlutaMAX ™ +HEPES	Cell Bank of Academia Sinica
		• 5%CO2, 37°C.	
C32TG	Melanoma	• Eagle’s minimal essential medium + FBS (10%)	JCRB Cell Bank
		• 5%CO2, 37°C.	
RT4	Papilloma of urinary bladder	• McCoy's 5a + FBS (10%)	DS Famer Biomedical
		• 5%CO2, 37°C	
TK	B-cell lymphoma	• RPMI + FBS (10%)	JCRB Cell Bank
		• 5%CO2, 37°C.	
UM-UC-14	Urothelial carcinoma of renal pelvis	• MEM・E (Glutamine(2 mM))+ NEAA (1%) + FBS (10%)	DS Famer Biomedical
		• 5%CO2, 37	

DMEM, Dulbecco's Modified Eagle Medium; HEPES, N-2-hydroxyethylpiperazine-N’-2-ethanesulfonic acid; FBS, fetal bovine serum; RPMI, Roswell Park Memorial Institute; MEM, Minimum Essential Medium; NEAA, non-essential amino acids; JCRB, Japanese Collection of Research Bioresources Cell Bank.

### Measurement of the cell membrane surface charge of fixed cells

#### Cell membrane surface charge measurement of cells in solution

The fixed cells were suspended in ultrapure water and the surface zeta potential was measured by Electrophoretic Light Scattering system (Malvern Panalytical, Malvern, UK).

#### Visualization of the cell membrane surface charge of each cell on glass slides

To visualize the cell membrane surface change of fixed cells, we used two different types of beads: 1) positively-charged and 2) antibody-labeled positively-charged magnetic beads (Dynabeads®, Life Technologies, Carlsbad, CA). The magnetic beads were made of iron oxide and the surface of the magnetic beads was treated with highly uniform polymer to make it hydrophilic.

#### Method used for the antibody-labeled beads

The Dynabeads® are commercially available beads, labeled with anti-rabbit antibodies. It was confirmed that there was no antigen-antibody cross-reactivity with human-derived cells, and that the antibodies were positively charged in a neutral solution. We mixed 50 μL of antibody-labeled beads and 1 mL of 0.1% bovine serum albumin (BSA) / PBS in a tube on DynaMag™-2 (Thermo Fisher scientific, Carlsbad, CA) for 2 minutes. After removing the supernatant, the same procedure was performed again. The mixture of the antibody-labeled beads and 1 mL of ultrapure water was added to the cultured cells at 4°C, for 12 hours.

#### Method used for the positively-charged beads

We prepared the positively-charged beads using the following procedure. First, we mixed 10 μL of beads (Bangs Laboratories, Fishers, IN) with 1 mL of 0.1% BSA / PBS in a tube on DynaMag™-2 for 2 minutes. After removing the supernatant, the same procedure was performed again. Then, the treated beads were mixed and stirred with White Slide Coat (Yuaikasei, Hyogo, Japan) on DynaMag™-2 for 1 minute. After the mixture was allowed to stand still for 2 minutes and the supernatant was removed, the positively-charged beads with 1 mL of ultrapure water were added to the cultured cells at 4°C, for 12 hours.

### Measurement of the cell membrane surface charge of each cell on glass slides

To measure the cell membrane surface charge of each cell on the glass slide, we counted the number of beads which were attached on the cell membrane surface. Since antibody-labeled beads showed a more stable positive charge and were more sensitive to subtle changes of the cell surface membrane charge than beads with positively-charged beads, we counted the number of beads on the fixed culture cells which reacted with the antibody-labeled beads. The fixed cells in the reaction solution, including the antibody-labeled beads, were filtered using a filter (Hologic Japan, Tokyo, Japan). After the addition of 1 mL of ultrapure water, 300 μL of the cell solution was used to prepare the cell specimen using an LBC precipitation method (Becton Dickinson, Franklin Lakes, NJ). The cell specimen was stained according to the Papanicolaou staining method. We counted the number of beads which were bound to 750 cells in each cell line.

### Effects of pH, salt concentration, and protein on the cell membrane surface charge

We assessed the effects of pH, salt, and protein in the reaction solution on the cell membrane surface charge. The assessments were made by counting the number of beads on fixed TK cells, in different reaction solutions with antibody-labeled beads. We prepared acidic and alkaline solutions and assessed the effect of pH on the charge of the membrane. The acidic solution was adjusted to pH 2.3 by adding 1 mL of acetic acid to 20 mL of ultrapure water (8.8×10^−4^mol/L), while the alkaline solution was adjusted to pH 13 by adding 1 mL of sodium hydroxide to 20 mL of ultrapure water (1.0×10^−4^mol/L). To assess the effect of salt concentration, we prepared a high salt concentration solution by adding twice the amount of NaCl to PBS (NaCl 1370 mM, KCl 27 mM, Na_2_HPO_41_12H_2_O 81 mM, KH_2_PO_4_ 18 mM) to make NaCl saturated. Also, we assessed the effect of protein using a 10% BSA solution. We counted the number of beads which were bound to 250 to 750 TK cells.

### Effect of cell membrane structure alterations on the cell membrane surface charge

To change the cell membrane structure, we treated fixed TK cells with 0.3% pepsin (sigma Aldrich, St. Louis, MO) / 0.01 M HCL. The assessments were performed by counting the number of antibody-labeled beads on fixed TK cells, which were treated by 0.3% pepsin at 40° C, for 30 seconds and 60 seconds. We counted the number of beads which were bound to 250 to 750 TK cells.

### Differences of the cell membrane surface charge of fixed cells among the different cell cycle phases and cell death

We determined the cell cycle status (interphase, prophase/ metaphase, anaphase and apoptotic) and cell death based on the nuclear morphology of fixed TK cells. We counted the number of antibody-labeled beads on 250 interphase cells, 70 prophase/ metaphase cells, 11 anaphase cells, and 70 apoptotic cells.

### Statistical analyses

For statistical analyses, a Wilcoxon test was applied using JMP Start Statistics version 14.1 (SAS Institute, Cary, NC).

## Results

### Cell membrane surface charge measurement in solution

**Fig 1:**
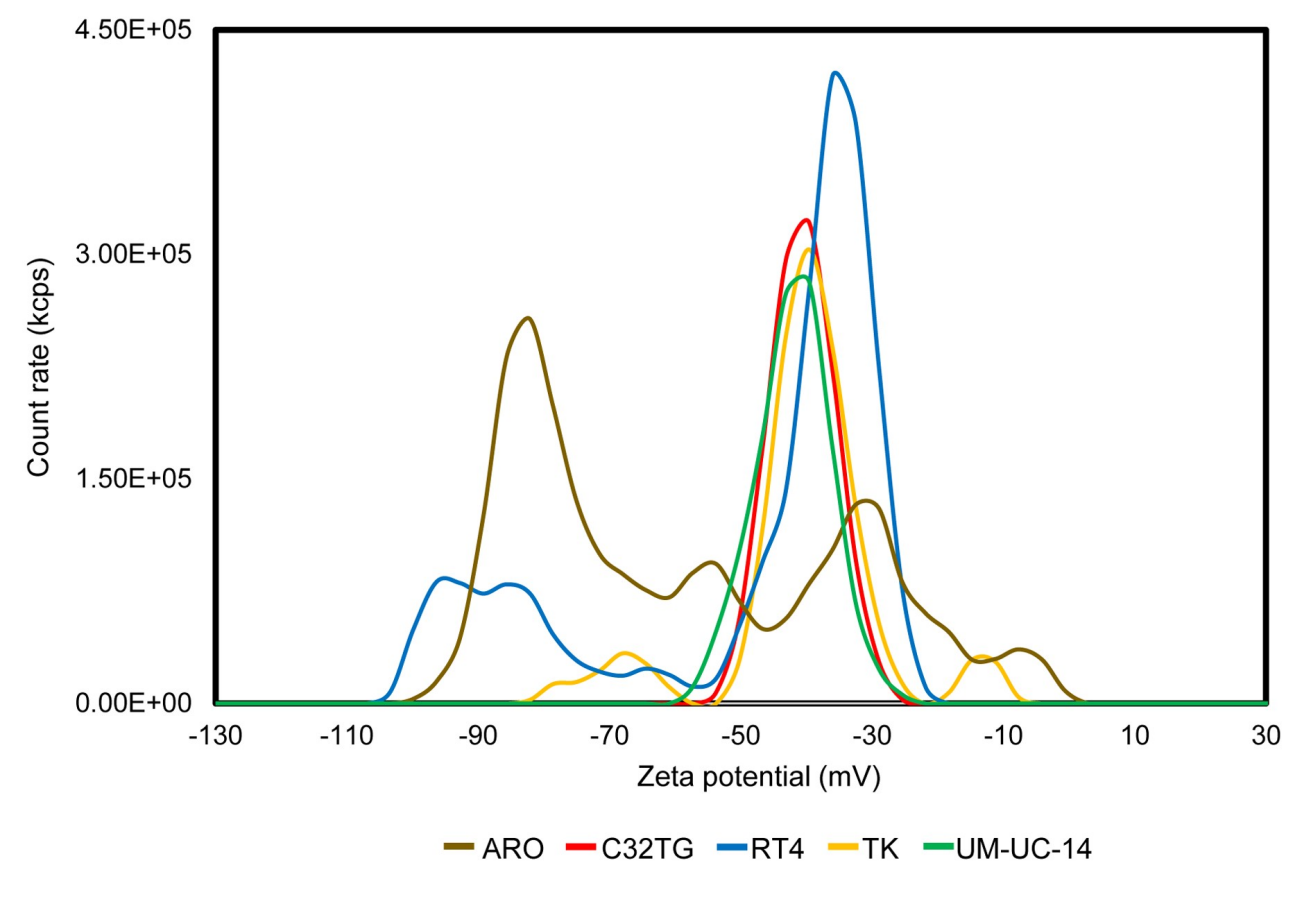
Zeta potential of fixed cells in several cell lines.

The electrophoretic light scattering analyzer showed that all of the cells fixed with Cytorich Red ™ were negatively charged (Fig 1). Among the fixed cells in each cell line, there were various cells with different zeta potential values. The average of three measurements and standard deviation of the zeta potential values of each cell line were -57.89 ± 22.63 mV on ARO cells, -40.41 ± 5.10 mV on C32TG cells, −46.99 ± 18.71 mV on RT4 cells, -40.13 ± 9.28 mV on TK cells, and −43.03 ± 5.52 mV on UM-UC-14 cells, respectively. As shown in Fig 1, monomodal and bimodal count rate peaks of cells with different zeta potential values were observed. In particular, C32TG cells and UM-UC-14 cells were monomodal and had a zeta potential of about -40 mV, whereas ARO cells, RT4 cells and TK cells were bimodal and had zeta potentials of about −40 mV and about −70 mV.

An electrophoretic light scattering analyzer showing that all the cells fixed with Cytorich Red ™ are negatively charged. Among the fixed cells in each cell line, there are various cells with different zeta potentials. Monomodal and bimodal count rate peaks of cells with different zeta potential values are observed. The averages and standard deviations of the zeta potential value of each cell line are as follows: -57.89 ± 22.63 mV on ARO cells, -40.41 ± 5.10 mV on C32TG cells, −46.99 ± 18.71 mV on RT4 cells, -40.13 ± 9.28 mV on TK cells, and −43.03 ± 5.52 mV on UM-UC-14 cells, respectively.

### Visualization of cell membrane surface charge of each cell on glass slides

**Fig 2:**
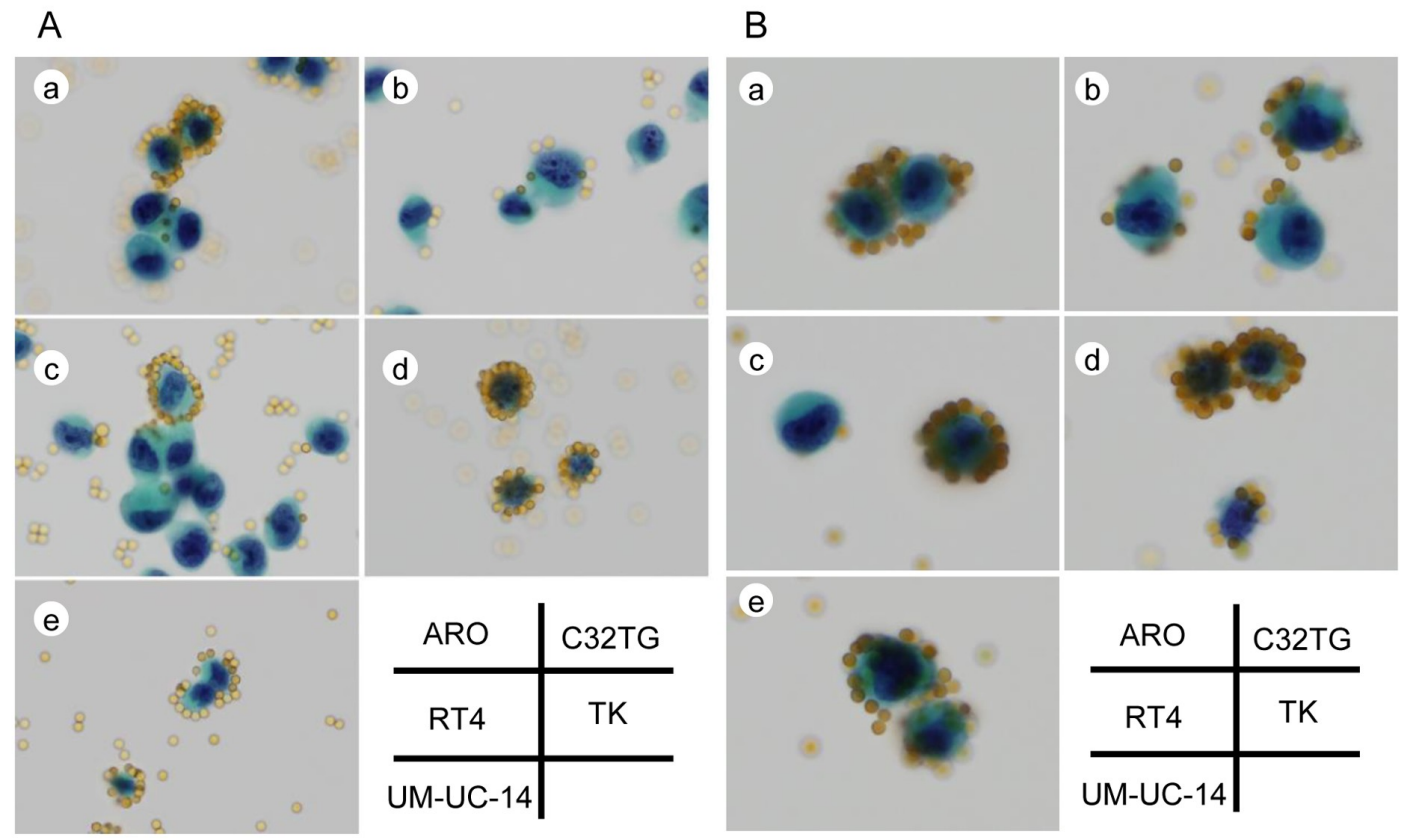
Visualization of the cell membrane surface charge of cells in 5 different cell lines.

**Fig 3:**
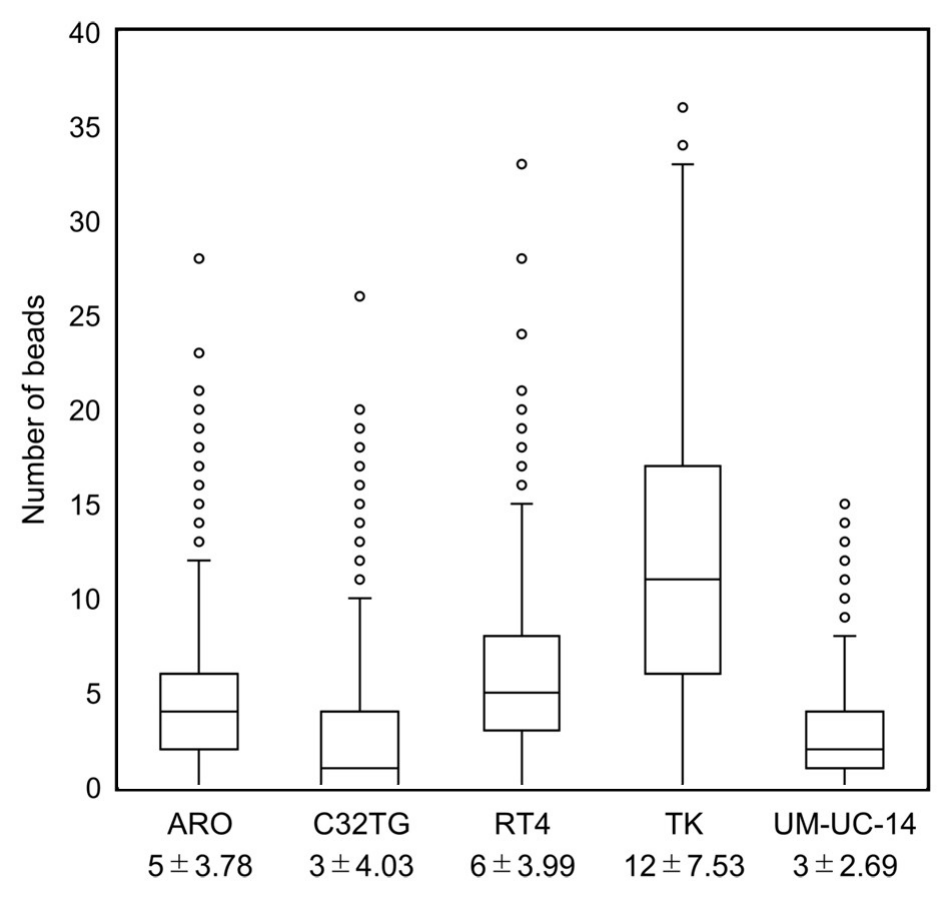
Scatter plot of the number of cell-bound antibody-labeled beads in 5 different cell lines.

We visualized the cell membrane surface charge of cells fixed with Cytorich Red ™ using antibody-labeled beads and positively-charged beads (Fig 2A and 2B). Binding of the beads to the fixed culture cells was observed at the cell membrane surface. Since both types of beads were positively changed, the binding observed indicated that the cell membrane surface of the fixed cells was negatively changed. The number of antibody-labeled beads that were bound to the cell membrane surface varied (0 to 36) among cells in the same cell line and among cells of different cell lines, since the beads were circumferentially or partially bound to the membrane surface with partial or whole petal-like pattern. The average and standard deviation of the number of antibody-labeled beads bound to each cell were 5 ± 3.78 for ARO cells, 3 ± 4.03 for C32TG cells, 6 ± 3.99 for RT4 cells, 12 ± 7.53 for TK cells and 3 ± 2.69 for UM-UC-14 cells (p<0.001) (Fig 3).

Representative images of the visualized cell membrane surface charge of fixed cells stained with the Papanicolaou stain, using antibody-labeled beads (A), and positively charged beads (B) (original magnification, x400). The beads are binding to the cell membrane surface, along the cell membrane contour, in the different cell lines (a: ARO, b: C32TG, c: RT4, d:TK and e: UM-UC-14). The distribution of the beads is circumferential or partial with a partial or whole petal-like pattern depending on the strength of the charge along the cell membrane contour.

A scatter plot showing the average and standard deviation of the number of cell-bound antibody-labeled beads in each cell in 5 different cell lines. We measured 750 cells in each cell line. The average and standard deviation of the number of antibody-labeled beads bound to each cell were 5 ± 3.78 for ARO cells, 3 ± 4.03 for C32TG cells, 6 ± 3.99 for RT4 cells, 12 ± 7.53 for TK cells and 3 ± 2.69 for UM-UC-14 cells (p<0.001).

### Effect of different fixatives on the cell membrane surface charge

**Fig 4:**
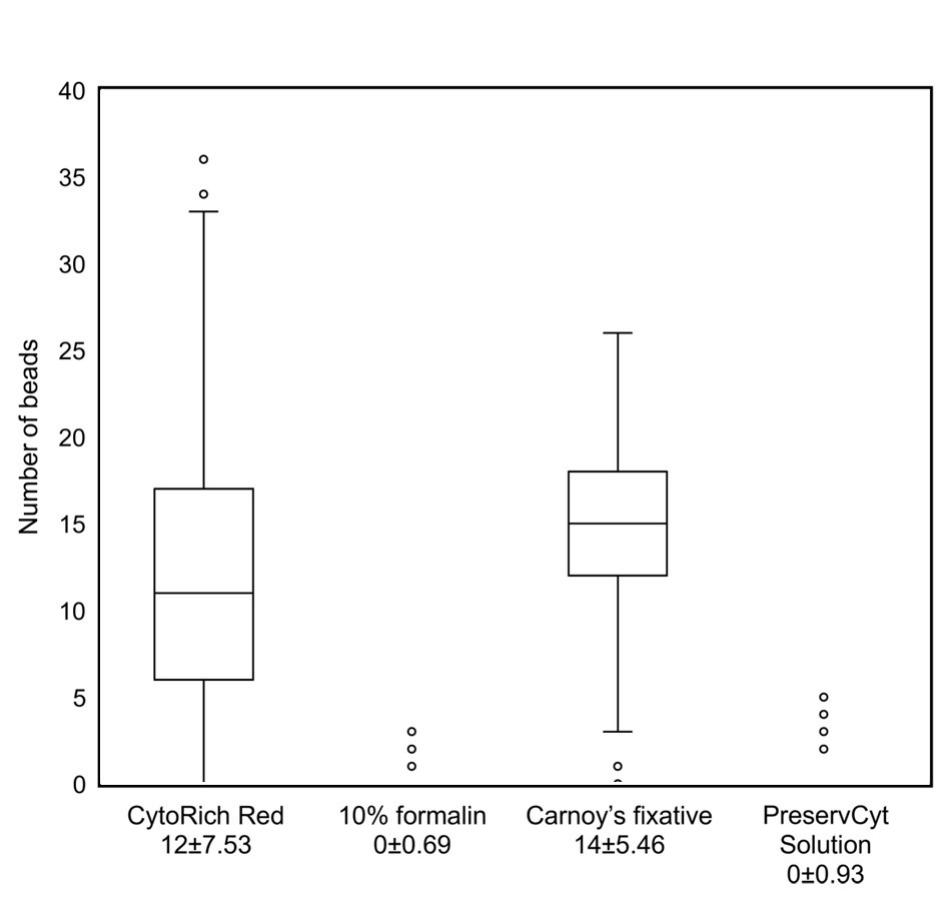
Scatter plot of the number of cell-bound beads in TK cells fixed with different fixatives.

The number of antibody-labeled beads bound to the cell membrane surface varied among TK cells fixed with different fixatives (CytoRich Red, 10% formalin, Carnoy’s fixative and PreservCyt Solution). The average and standard deviation of the number of beads per cell were 12 ± 7.53 in cells fixed with Cytorich Red ™, 0 ± 0.69 in cells fixed with a 10% formalin solution, 14 ± 5.46 in cells fixed with Carnoy's and 0 ± 0.93 in cells fixed with PreservCyt Solution (p<0.001) (Fig 4).

A scatter plot shows the number of cell-bound antibody-labeled beads in TK cells fixed with different fixatives. The average and standard deviation of the number of beads per cell were 12 ± 7.53 in cells fixed with Cytorich Red ™, 0 ± 0.69 in cells fixed with a 10% formalin solution, 14 ± 5.46 in cells fixed with Carnoy's and 0 ± 0.93 in cells fixed with PreservCyt Solution (p<0.001).

### Effect of pH, salt and protein concentration on cell membrane charge

**Fig 5:**
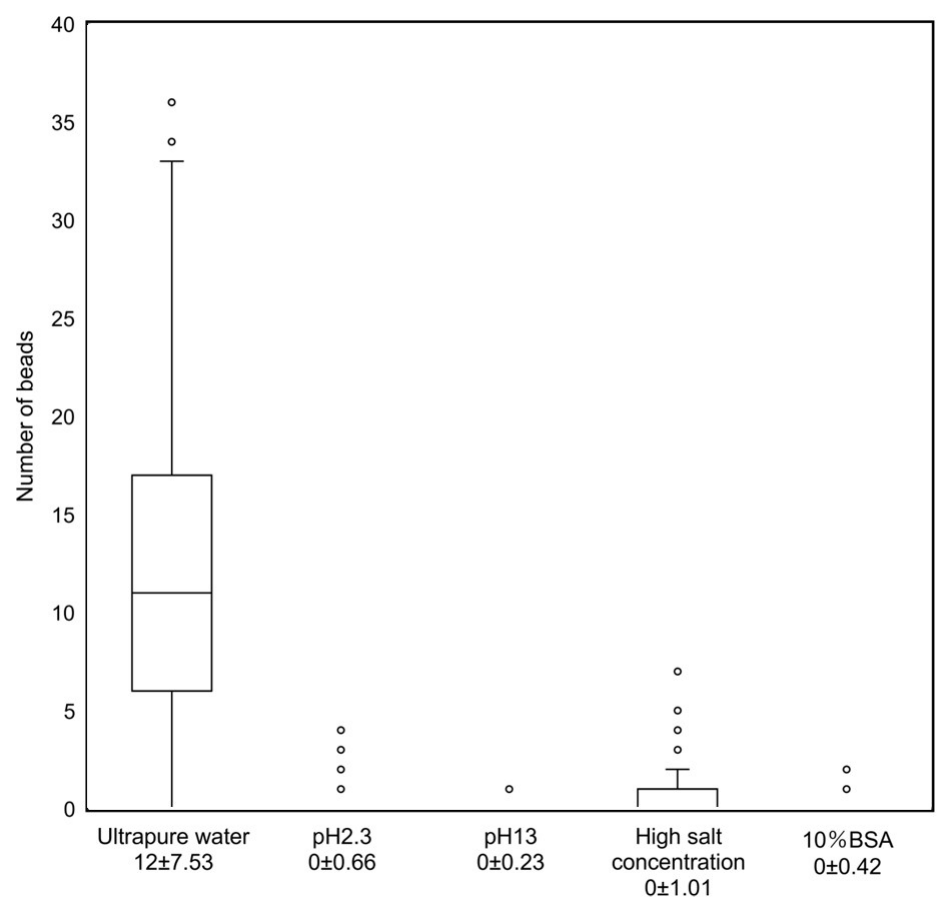
Scatter plot of the number of cell-bound beads in fixed TK cells in different solutions.

The number of antibody-labeled beads bound to the cell membrane surface varied among TK cells in solutions with different pH, different salt and BSA concentration. The average and standard deviation of the number of beads bound per cell were 0 ± 0.66 in cells in a pH 2.3 solution, 0 ± 0.23 in cells in a pH 13 solution, 0 ± 1.01 in cells in a high salt concentration solution, and 0 ± 0.42 in cells in a 10% BSA solution (p<0.001) (Fig 5).

A scatter plot shows the number of cell-bound antibody-labeled beads in fixed TK cells, in solutions with different pH, salt and BSA concentration. The average and standard deviation of the number of beads per cell were 0 ± 0.66 in cells in a pH 2.3 solution, 0 ± 0.23 in cells in a pH 13 solution, 0 ± 1.01 in cells in a high salt concentration solution, and 0 ± 0.42 in cells in a 10% BSA solution (p<0.001).

### Effect of cell membrane structure change on the cell membrane surface charge

**Fig 6:**
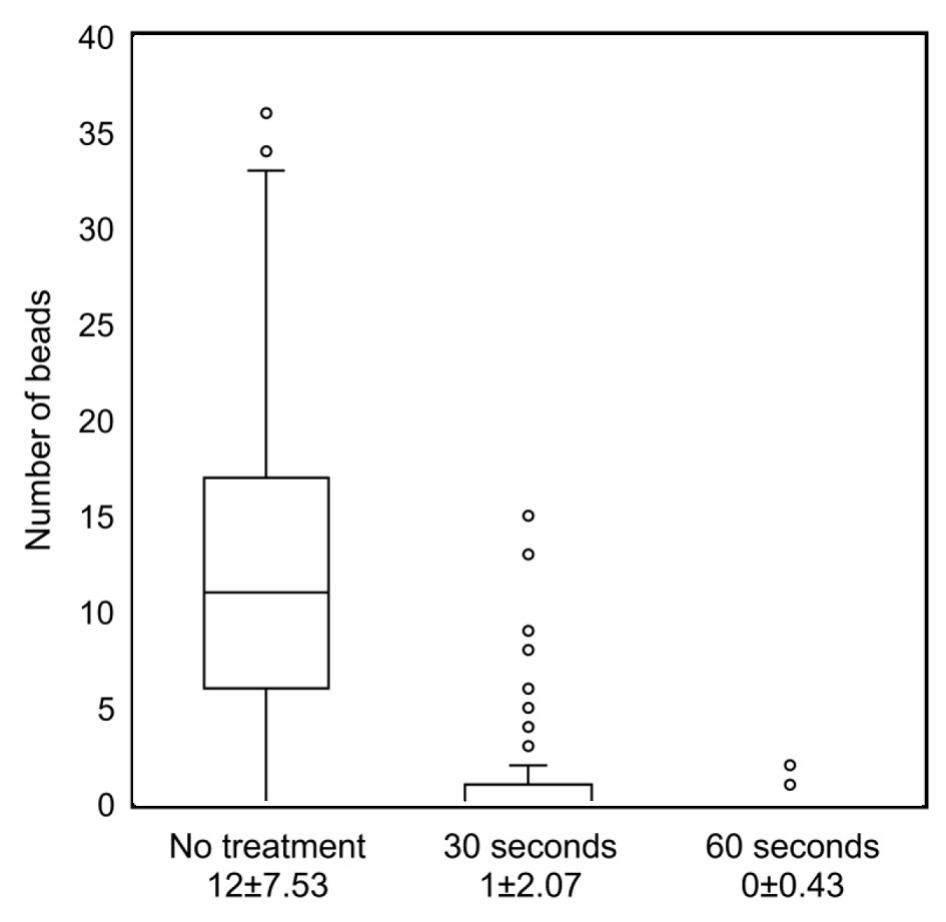
Scatter plot of the number of cell-bound beads in fixed TK cells treated with pepsin.

Fixed TK cells were treated with pepsin to change cell membrane structure. The number of antibody-labeled beads decreased depending on the reaction time. The average and standard deviation of the number of beads per cell were 1 ± 2.07 in cells in a reaction that lasted for 30 seconds and 0 ± 0.43 for those in a reaction that lasted for 60 seconds (p<0.0001) (Fig 6).

Scatter plot indicates the number of cell-bound antibody-labeled beads in fixed TK cells treated with a 0.3% pepsin solution. The average and standard deviation of the number of beads per fixed TK cell were 1 ± 2.07 in cells in a reaction that lasted for 30 seconds, and 0 ± 0.43 in a reaction that lasted for 60 seconds (p<0.0001).

### Differences of cell membrane surface charge of fixed cells in different cell cycle phases and dead cells

**Fig 7:**
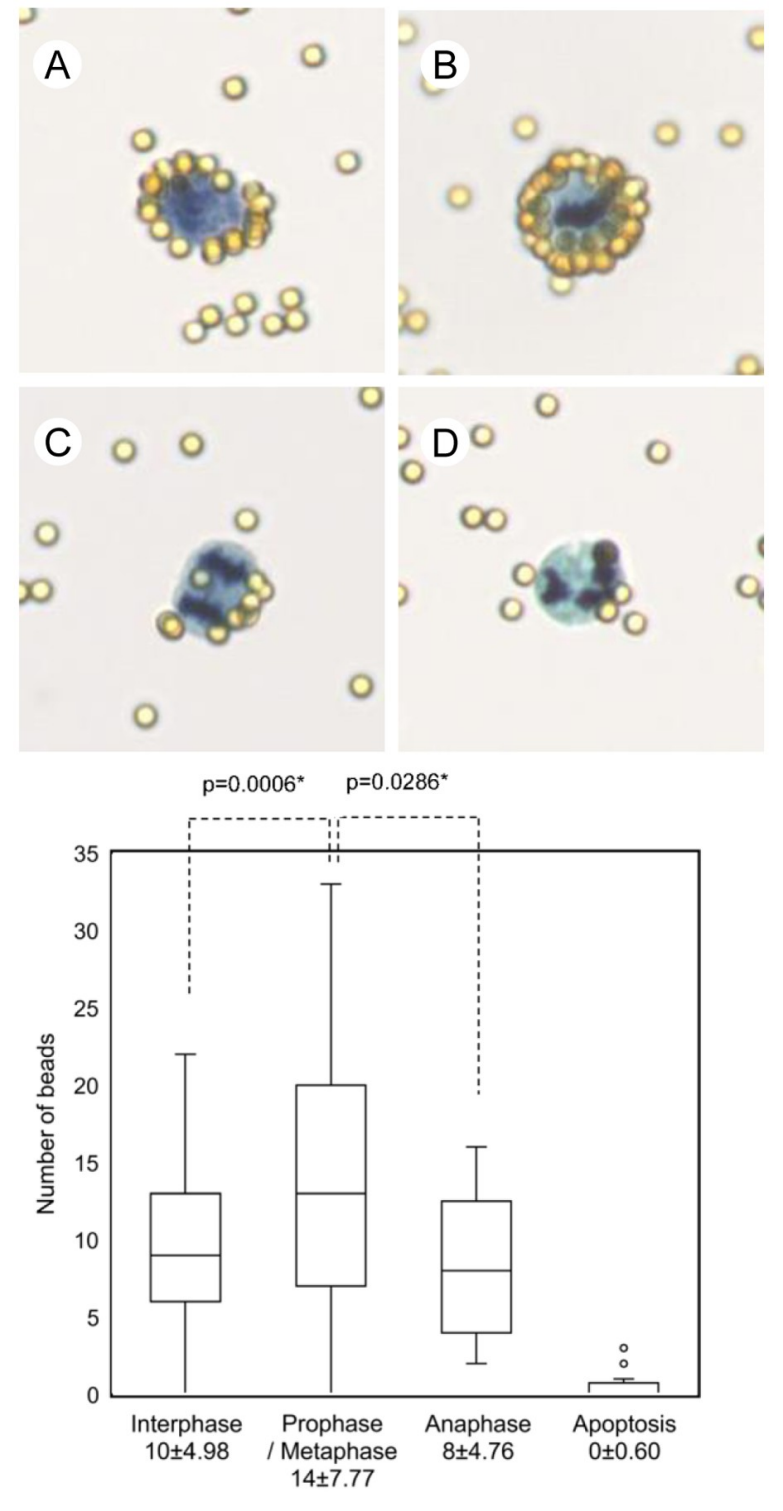
Visualization of cell membrane surface change and scatter plot in different cell status.

The number of antibody-labeled beads bound with fixed TK cells in anaphase was smaller than the numbers observed in any other cell cycle phase. The average number and standard deviation of the number of beads per cell were 10 ± 4.98 in interphase cells, 14 ± 7.77 in prophase / metaphase cells, and 8 ± 4.76 in anaphase cells, respectively (p = 0.0286). We detected almost no beads bound with apoptotic cells (Fig 7).

Visualization and scatter plot of cell-bound antibody-labeled beads in different cell cycle stages and apoptotic cells in the fixed TK cells are shown. Representative images of the visualized cell membrane surface charge of fixed cells in interphase (A), prophase / metaphase (B), anaphase (C) and apoptotic phase (D), in fixed TK cells stained with the Papanicolaou stain (original magnification, x400). The average number and standard deviation of the number of beads per cell were 10 ± 4.98 in interphase cells, 14 ± 7.77 in prophase / metaphase cells, and 8 ± 4.76 in anaphase cells, respectively. Almost no beads bound to apoptotic cells.

## Discussion

Cell proliferation in cancer is associated with a high membrane potential in living cells due to activation of potassium ion channels [14, 15]. The membrane potential of living cancer stem cells in hepatocellular carcinoma has a lower negative charge than that of normal stem cells [16]. Thus, it has been confirmed that the membrane potential of living cells involves various cellular biological activities [17–20]. However, fixed cells lose the dynamics of the membrane potential, since the intracellular metabolism with ion dynamics ceases to exist. Considering a previous report of the relation between the cell membrane surface charge of erythrocytes and the distribution and density of sialic acid in cell membrane [21, 22], we hypothesized that fixed cells still have a cell membrane surface charge due to the presence of structural proteins and lipids on the cell membrane. Thus, the charge state of the cell membrane surface of fixed cells could be affected by the structure of the cell membrane surface, which is related with cell differentiation, cell proliferation, and malignant potential.

Although methods exist to measure the membrane potential of living cells, using micro-pipettes and scanning ion conductance microscopy [23, 24] and dyes such as DiBAC_4_ [25], they cannot be applied to measure the cell membrane surface charge of fixed cells. However, an electrophoretic light scattering system can measure the surface zeta potential on various particles in a liquid solution based on their electrophoretic mobility [26–29]. In this study, the surface zeta potentials of all the fixed cells were negative. Furthermore, the zeta potential values varied among the different cell lines and among cells of the same cell line. To analyze the relationship between cell membrane surface charge and cell morphology, we needed to develop methods to visualize the cell membrane surface charge. Bingdi Chen et al. visualized membrane potential using positively charged beads in living cells [26]. In this study, we visualized the cell membrane surface charge of each fixed cell using both antibody-labeled beads and positively-charged beads. Since antibodies are positively charged in ultrapure water, the antibody-labeled beads used in this study bound to the negatively charged cell membrane surface in a similar way as positively-charged beads did.

Consistent with the results of zeta potential measurements of fixed cells, our visualizing methods using both positively-charged beads and antibody-labeled beads, proved that the membrane surface of fixed cells was negatively charged. The results showed no obvious differences between positively charged beads and antibody-labeled beads. However, the results of measurements of zeta potentials and visualized beads were different regarding the cell membrane surface charge of fixed cells. We hypothesized that the difference was related to whether the cells were floating as spheres or attached to a glass slide, in a flat state. Moreover, the zeta potential only accounts for the charge on the cell membrane surface, whereas the antibody-labeled beads also reflected the three-dimensional structure of proteins on the cell membrane surface. As shown in Fig 2, the distribution of beads, namely, the cell membrane surface change, varied depending on the strength of the charge on the different areas along the cell membrane contour of a fixed cell. By visualizing the cell membrane surface charge, we could detect variations of the cell membrane surface charge associated with cell morphology.

As mentioned above, we hypothesized that by using antibody-labeled beads we could sensitively visualize the biological state of the cell membrane surface, which is affected by the three-dimensional structure of proteins as well as the different types of amino acids and lipids. Furthermore, alternations of the three-dimensional structure of proteins on the cell membrane surface by different fixative solutions and pepsin treatment, resulted in a change of the cell membrane surface charge. Moreover, the number of beads bound to the cell membrane surface was smaller in solutions of different pH and ion concentration than in ultrapure water, since the solutions affected the electrophoretic mobility. The above results indicated that the binding between the cell membrane surface and antibody-labeled beads was caused by positive-negative charge interactions, rather than by an antigen-antibody reaction. Thus, the results we obtained in this study proved our hypothesis.

Cell morphology varies among the different cell types and also depends on a cell’s differentiation state, processes such as proliferation, and malignant potential. The cell morphology of the different cell types may be related to cell membrane components, such as different proteins and lipids. Thus, we suggest that an assessment of the cell membrane surface charge may reveal the biological characteristics of the cells. For example, cancer cells with an abnormal morphology have a higher density of sialic acid in their cell membranes than normal cells with normal morphology [30]. Moreover, it has been reported that living cells change cell membrane components or characteristics, such as cell membrane proteins and membrane potential during the cell cycle [31]. Consistent with a previous report where living cells were used, our results in fixed cells showed changes in the number of cell-binding beads, representing the cell membrane surface charge, during the cell cycle. As our results demonstrated, the number of beads bound to interphase and anaphase cells was smaller than the number of beads bound to prophase or metaphase cells, while a small number of beads bound to apoptotic cells. These changes in the cell membrane surface charge suggest that the cell membrane structure changes during the cell cycle and cell death.

In conclusion, to the best of our knowledge, this is the first systematic report to prove that the cell membrane surface of fixed cells is negatively charged, using visualization methods with both positively-charged beads and antibody-labeled beads, as well as zeta potential measurements. Moreover, the charge state on the cell membrane surface of fixed cells was affected from the amino acid composition and three-dimensional structure of proteins on the cell membrane surface. Our results indicate that an assessment of the cell membrane surface charge of fixed cells can be useful when we aim to determine the biological characteristics of different types of cells or to differentiate between cells in a different cell state, cell cycle stage, or malignant potential, etc. Therefore, we conclude that we will be able to apply these methods to visualize the cell membrane surface charge of fixed cells to aid clinical cytological diagnosis, and establish a differential diagnosis between benign and malignant cells.
